# Additive and Interactive Genetically Contextual Effects of HbA1c on cg19693031 Methylation in Type 2 Diabetes

**DOI:** 10.3390/genes13040683

**Published:** 2022-04-13

**Authors:** Kelsey Dawes, Willem Philibert, Benjamin Darbro, Ronald L. Simons, Robert Philibert

**Affiliations:** 1 Department of Psychiatry, University of Iowa, Iowa City, IA 52242, USA; willem-philibert@uiowa.edu (W.P.); robert-philibert@uiowa.edu (R.P.); 2 Department of Pediatrics, University of Iowa, Iowa City, IA 52242, USA; benjamin-darbro@uiowa.edu; 3 Department of Sociology, University of Georgia, Athens, GA 30602, USA; rsimons@uga.edu; 4 Behavioral Diagnostics LLC, Coralville, IA 52246, USA; 5 Cardio Diagnostics Inc., Coralville, IA 52246, USA

**Keywords:** epigenetics, DNA methylation, type 2 diabetes

## Abstract

Type 2 diabetes mellitus (T2D) has a complex genetic and environmental architecture that underlies its development and clinical presentation. Despite the identification of well over a hundred genetic variants and CpG sites that associate with T2D, a robust biosignature that could be used to prevent or forestall clinical disease has not been developed. Based on the premise that underlying genetic variation influences DNA methylation (DNAm) independently of or in combination with environmental exposures, we assessed the ability of local and distal gene x methylation (GxMeth) interactive effects to improve cg19693031 models for predicting T2D status in an African American cohort. Using genome-wide genetic data from 506 subjects, we identified a total of 1476 GxMeth terms associated with HbA1c values. The GxMeth SNPs map to biological pathways associated with the development and complications of T2D, with genetically contextual differences in methylation observed only in diabetic subjects for two GxMeth SNPs (rs2390998 AG vs. GG, *p* = 4.63 × 10^−11^, Δ*β* = 13%, effect size = 0.16 [95% CI = 0.05, 0.32]; rs1074390 AA vs. GG, *p* = 3.93 × 10^−4^, Δ*β* = 9%, effect size = 0.38 [95% CI = 0.12, 0.56]. Using a repeated stratified k-fold cross-validation approach, a series of balanced random forest classifiers with random under-sampling were built to evaluate the addition of GxMeth terms to cg19693031 models to discriminate between normoglycemic controls versus T2D subjects. The results were compared to those obtained from models incorporating only the covariates (age, sex and BMI) and the addition of cg19693031. We found a post-pruned classifier incorporating 10 GxMeth SNPs and cg19693031 adjusted for covariates predicted the T2D status, with the AUC, sensitivity, specificity and precision of the positive target class being 0.76, 0.81, 0.70 and 0.63, respectively. Comparatively, the AUC, sensitivity, specificity and precision using the covariates and cg19693031 were only 0.71, 0.74, 0.67 and 0.59, respectively. Collectively, we demonstrate correcting for genetic confounding of cg19693031 improves its ability to detect type 2 diabetes. We conclude that an integrated genetic–epigenetic approach could inform personalized medicine programming for more effective prevention and treatment of T2D.

## 1. Introduction

Type 2 diabetes mellitus (T2D) is the seventh leading cause of morbidity and mortality in the US and is responsible for $327 billion annually in economic damage [1]. T2D is diagnosed in the presence of sustained hyperglycemia that is above the threshold that predisposes to microvascular complications [2]. However, hyperglycemia is the end product of several pathophysiological processes that eventually converge on the inability of the pancreatic β cells to produce enough insulin to meet the demands of target tissues. Our inability to identify and adequately understand this etiological heterogeneity underpins the diagnostic and prognostic challenges faced in clinical practice. Thus, novel precision diagnostic methods that refine the characterization of diabetes to optimize prognostication and therapies are highly desired to improve clinical decisions and patient outcomes.

T2D has a complex genetic and environmental architecture that underlies its development and clinical presentation [3]. Genome-wide association studies (GWAS) have identified more than 500 common genetic variants associated with the disease. However, in aggregate, these explain less than 20% of the heritability [4]. Other attempts to elucidate the disease etiology include identifying epigenetic alterations that mediate environmental influences on aberrant gene expression. DNA methylation (DNAm) is the most widely studied and characterized epigenetic modification due to its scalability and its potential for clinical translation. Several investigators have demonstrated that the DNAm status at cg19693031 is highly correlated with both HbA1c values and T2D [5,6,7]. However, these prior studies failed to account for potential confounding genetic influences. Given the dynamic cross-talk between genetic variation, DNA methylation and environmental exposures, these factors should not be considered as self-standing layers of pathophysiological regulation but should only be interpreted in light of each other [8]. For example, several genetic variants for common complex diseases are not detected in GWAS analyses when the environmental influences are not included. Thus, this partially informed approach of traditional GWAS/EWAS analyses fails to adequately explain the interconnected network that underlies the development and progression of diabetes, which limits our ability to prevent and treat the disease.

In this communication, we use an omni-genic approach in an attempt to explain how the interactions between cg19693031 methylation and common variants in the cellular context of hyperglycemia can increase disease susceptibility. We then implement functional analyses to investigate possible mechanisms explaining the association between the methylation status of TXNIP’s 3′UTR and prevalent T2D. Finally, we examine if correcting for genetic confounding improves the ability of cg19693031 to detect T2D.

## 2. Materials and Methods

### 2.1. Subjects and Recruitment

This study uses genomic and biological data from Wave 5 of primary caregivers (PC) of the Family and Community Health Study (FACHS). The overall design and methods used in the FACHS study have been described previously [9]. Each of these procedures was approved by the University of Iowa Institutional Review Board (IRB 200802719). The data from a total of 506 subjects were retained for further analyses.

DNA was prepared using standard cold protein precipitation [10]. Glycosylated hemoglobin A_1c_ (HbA1c) levels were determined by the University of Iowa Diagnostic Laboratories (https://medicine.uiowa.edu/uidl/, accessed on 4 February 2018) using turbidimetric immune inhibition [11] in compliance with standard Clinical Laboratory Improvement Amendments (CLIA) procedures [12].

### 2.2. Genome-Wide DNA Methylation

Genome-wide methylation was determined by the University of Minnesota Genome Center (http://genomics.umn.edu/, accessed on 10 March 2018) using the Infinium MethylationEpic Beadchip (Illumina, San Diego, CA, USA) according to the manufacturer’s protocol. Standard sample and probe level quality control of the resulting data were conducted as previously described [13].

### 2.3. Genome-Wide Genotypes

Genome-wide genotype information also was determined by the University of Minnesota Genome Center using the Multi-Ethnic Global-8 Beadchip (Illumina, San Diego, CA, USA) according to the manufacturer’s suggested protocol. Quality control was performed at both the sample and single nucleotide polymorphism (SNP) probe levels in PLINK [14]. SNP probes with a minor allele frequency >5% were retained. Linkage disequilibrium-based SNP pruning was performed with a window size of 50 SNPs, window shift of 5 SNPs and a pairwise SNP-SNP LD threshold of 0.5. A total of 516908 variants for 506 subjects passed filters and QC. For the purposes of these analyses, genotypes were recoded as 0, 1 or 2 in PLINK.

Genotypes at rs7211, rs7212 and rs9245 were determined by our standard primer probe genotyping procedures using fluorescent hydrolysable probe assays and reagents from ThermoFisher (Waltham, MA, USA) according to manufacturer’s instructions [15].

### 2.4. Phenotypes

Phenotypes that were considered in the analyses include age, gender, body mass index (BMI) and type 2 diabetes mellitus (T2D) status. The age used was the age of the subject at the time of the Wave 5 interview. Gender was coded as a binary variable representing self-reported biological sex. BMI was calculated using the measured height and weight of the subject (kg/m^2^) and used as a continuous variable.

T2D status was determined for each subject by binning the HbA1c value at the time of the W5 interview. The binning criteria were adapted from the clinical diagnostic guidelines set by the American Diabetes Association; i.e., those with an HbA1c ≤ 5.7 were binned as normoglycemic controls, HbA1c between 5.7–6.5 were binned as pre-diabetic and HbA1c ≥ 6.5 as diabetic [2]. Normoglycemic control subjects self-reporting a T2D diagnosis and any non-diabetic subject self-reporting the use of anti-diabetic pharmacotherapy were removed from the final dataset.

### 2.5. Identification of HbA1c-Associated DNAm Loci

The identification of DNAm loci associated with HbA1c values was determined by fitting a linear regression model in R v.3.1.2 using the following equation:

$$HbA1c\;\sim \;Meth_{i}+Age+Sex+BMI$$

A total of 861,916 independent analyses were conducted. The DNAm loci with a genome-wide significance after FDR correction (α = 0.05) were retained for further analyses.

### 2.6. Identification of Stochastic Epimutations

The distribution and variability of methylation values were evaluated using boxplots for each of the identified HbA1c associated DNAm loci. Stochastic epimutations (SEMs) were defined as methylation outliers exceeding three times the interquartile range (IQR), consistent with the definition of Gentilini and colleagues [16]. SEMs were further classified based on the methylation value compared to the average of the population, as previously described by Wang and associates [17]. High methylation outliers (HMO) were defined using the following equation:

HMO=Q3+(3×IQR)

Similarly, low methylation outliers (LMO) were defined using the following equation:

LMO=Q1−(3×IQR)

HbA1c-associated DNAm probes containing SEMs were removed from further analyses.

### 2.7. Identification of HbA1c-Associated Polymorphic CpGs

DNAm probes potentially affected by polymorphisms at the target site were identified using methods previously described. In brief, the Infinium MethylationEPIC BeadChip manifest file was used to identify SNPs found at the target site for both Infinium Type I and Type II probes. To identify those associated with HbA1c values, a linear regression model was fit in R using the following equation:

$$HbA1c\;\sim \;Meth_{i}+Age+Sex+BMI$$

A total of 302,943 independent analyses were conducted. The DNAm loci with a genome-wide significance after FDR correction (α = 0.05) were retained for further analyses.

### 2.8. Identification of meQTL Effects of cg19693031 Methylation

To understand the genetically contextual effects on cg19693031 methylation, meQTL analyses in both cis and trans were conducted. Linear regression models were fit in R using the following equation:

$$Meth\;\sim \;SNP_{i}+Age$$

A total of 117 independent cis analyses of potential interaction effects with cg19693031 were conducted. Further, meQTLs acting in cis were defined as SNPs located on chromosome 1 and within 1Mb of cg19693031. A total of 38750 independent long-range cis analyses were conducted, defined as SNPs located on chromosome 1 and located > 1 Mb of cg19693031. A total of 459464 independent trans analyses were conducted, defined as SNPs not residing on chromosome 1. SNPs with nominally significant effects were retained for further analyses (α = 0.05).

### 2.9. Genetic + Environmental (G + E) Analyses

The additive genetic and environmental effects on cg19693031 methylation were assessed using the following equation in R:

$$Meth\;\sim \;SNP_{i}+SNP_{j}+HbA1c+Age$$

SNPs with nominally significant effects were retained for further analyses (α = 0.05).

### 2.10. Genetic x Environment (GxE) Analyses

The genetically contextual effects of HbA1c on cg19693031 methylation were assessed using the following equation in R:

$$Meth\;\sim \;SNP_{i}\times HbA1c+SNP_{i}+HbA1c+Age$$

In particular, the interaction term *p*-value was used for the selection and ranking of SNPs with nominally significant GxE effects. A total of 117, 38,750 and 459,464 independent cis, long-range cis and trans analyses were conducted, respectively.

### 2.11. Local TXNIP GxMeth Logistic Regression Classifiers for T2D Status

Training and test datasets were prepared to develop and evaluate each classifier for T2D status in Python v.3.8.10 [18]. A total of 137, 260 and 90 normoglycemic controls, prediabetic and diabetic subjects were retained for logistic modeling. These subjects were used to generate the training (70%) and testing (30%) datasets. T2D status was coded as binary variable, with the more severe dysglycemic status reflecting the positive target class. The target class was stratified between the training and testing datasets to reflect the original class ratio. The performance of each model was evaluated using the receiver operating characteristic (ROC) area under the curve (AUC), sensitivity, specificity and precision of the more severe dysglycemic target class.

### 2.12. Identification of Distal GxMeth Interactions

The identification of HbA1c-associated distal significant Genetic x Methylation (GxMeth) interactions was determined by fitting a linear regression using the following equation in R:

$$HbA1C\;\sim \;cg19693031\times SNP_{i}+SNP_{i}+cg19693031+Age+Sex+BMI$$

Additive and interactive GxMeth terms were evaluated for each SNP that survived data cleaning and filtering, with a total of 614,729 independent analyses conducted. The SNPs of each GxMeth interactive term with genome-wide significance after Bonferroni correction (α = 0.05) were retained for further analyses. The R package *CMplot* was used to construct the Manhattan plot [19].

### 2.13. Distal GxMeth Functional SNP Mapping

Each SNP with a significant GxMeth interaction term was annotated using the Multi-Ethnic Global-8 Annotation File. The 560 mapped genes were used to generate a protein–protein interaction (PPI) network using the STRING database [20]. To investigate the functions of the GxMeth genes, functional enrichment analysis was performed using gene ontology (GO) and Kyoto Encyclopedia of Genes and Genomes (KEGG) [21].

### 2.14. Integrated Genetic–Epigenetic Balanced Random Forest Classifier for T2D Status

A series of balanced random forest classifiers (BRF) were built using *scikit-learn* in Python v.3.8.10 to evaluate the predictive ability of integrated GxMeth models for discriminating normoglycemic controls versus diabetic subjects. Each BRF classifier was built using a repeated stratified cross-validation (10 splits and 3 repeats) approach. The dimensionality of the dataset was reduced by taking advantage of the implicit feature selection of the random forest using the *SelectFromModel* function in *sklearn*. A BRF classifier was built as previously described to evaluate the selected features for predicting T2D status (normoglycemic controls versus diabetic subjects). The selected features were then ranked according to their Gini importance, with the top 13 features retained.

## 3. Results

### 3.1. Clinical and Demographic Characteristics

The key demographic and clinical characteristics of the 506 subjects are given in Table 1. The subjects in each group were almost exclusively African American, and predominantly female. Congruent with the clinical observations, the diabetic subjects were significantly older and more obese than the normoglycemic and prediabetic subjects (*p* = 1.29 × 10^−9^ and *p* = 1.12 × 10^−6^, respectively). While the systolic and diastolic blood pressures were not significantly different between the groups, there was a higher prevalence of self-reported hypertension and anti-hypertensive therapy in the diabetic subjects. A total of 73 subjects self-reported a history of diabetes, with 70% of these subjects having an HbA1c ≥ 6.5% despite only 4% reporting anti-diabetic pharmacological treatment.

### 3.2. Identification of HbA1c-Associated DNA Methylation Loci

**Figure 1:**
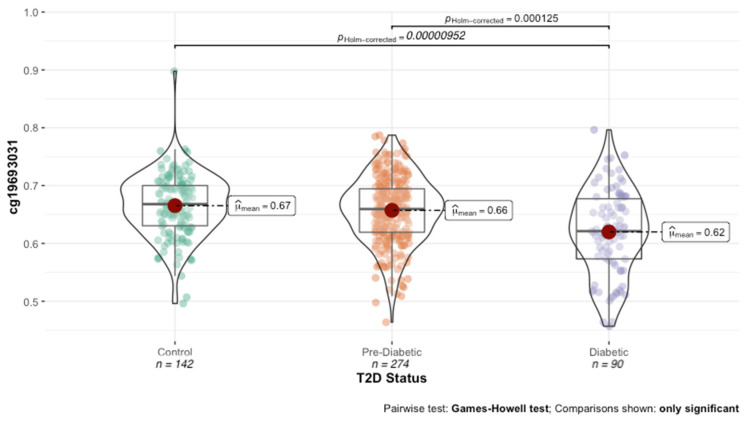
Distribution of cg19693031 methylation across T2D status.

A total of 514,292 methylation probes survived the data cleaning and quality control measures. To identify HbA1c-associated DNAm loci, each probe was regressed against HbA1c values, controlling for age, sex and BMI. A total of 23 DNAm probes were associated with HbA1c at a false-discovery rate (FDR) of <5% (Table 2). Consistent with prior studies, cg19693031 was the most highly associated probe (R^2^ = 0.1976, *p* = 4.43 × 10^−12^). As illustrated in Figure 1, cg19693031 was significantly demethylated in diabetic subjects compared to both normoglycemic controls (62% vs. 66%; t = 5.089, *p* = 1.06 × 10^−6^) and prediabetic subjects (62% vs. 66%; t = −4.4165, *p* = 2.12 × 10^−5^).

**Figure 2:**
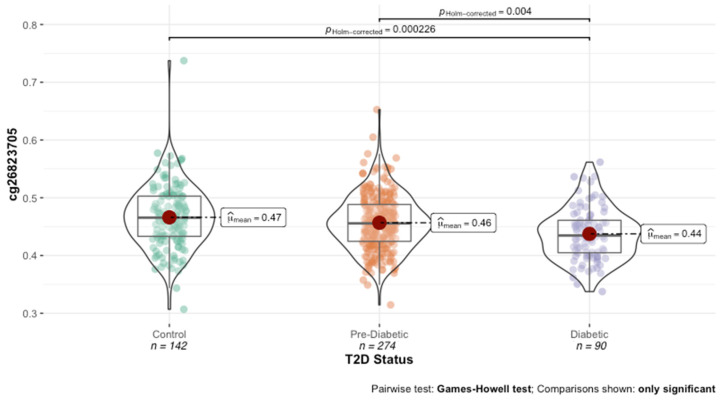
Distribution of cg26823705 across T2D status.

The distribution and variability of the methylation values were evaluated for each of the identified HbA1c-associated DNAm loci. With the exception of cg19693031 and cg26823705 (Figure 2), the associations to HbA1c were driven by outliers. Consistent with the definition by Gentilini and colleagues, a total of 49 stochastic epimutations (SEMs) were identified in 27 subjects across the 23 DNAm loci. With the exception of cg1963031, SEMs were observed in each probe. The SEMs were significantly enriched in diabetic subjects (OR = 0.135, 95% CI = 0.072–0.248, *p*-value = 1.40 × 10^−11^, two-tailed Fischer’s exact test), with 30 of the 49 total number of SEMs found in diabetic subjects. Further, six subjects had more than one SEM across the twenty-three probes, four of whom were diabetic.

The SEMs were further classified into two groups based on the methylation value compared to the population. A total of 26 SEMs were hypermethylated and classified as high methylation outliers (HMO), and a total of 23 SEMs were hypomethylated and classified as low methylation outliers (LMO). Each of the 22 probes contained either HMOs (*n* = 11) or LMOs (*n* = 11); however, none contained both. Notably, 14 (54%) and 23 (70%) of the identified HMOs and LMOs were observed in diabetic subjects.

To further characterize the HMOs and LMOs, we examined their locations in relation to CpG island regions. The majority of DNAm loci with HMOs resided in CpG islands (*n* = 7), with the remainder in open seas (*n* = 3). Notably, the Hidden Markov Model predicts an additional three of these loci are located within an island. Comparatively, the majority of DNAm loci with LMOs resided in the open sea (*n* = 8), with the remainder in subsequent shores (*n* = 1), shelves (*n* = 1) and islands (*n* = 1). These findings are consistent with those previously reported by Wang et al. [17]. All the DNAm loci with SEMs were excluded from further analyses.

A total of 37009 SEMs were detected in 64% (*n* = 13,528) of the 21269 HbA1c-associated DNAm probes with nominal significance. The total number of SEMs per probe ranged from 0 (*n* = 7740) to 102 across the 506 subjects. Of the 13,528 probes with SEMs detected, a majority (84%) contained less than five SEMs across the cohort. Additionally, a majority of the SEMs detected were HMOs (*n* = 25,620). The total number of SEMs per subject ranged from 3 to 7271 across the 21,269 probes, with a median of 20 ± 410 SEMs per subject. No relationship was observed in the number of SEMs with T2D status, age, gender or BMI. With the exception of cg19693031 and cg26823705, the association of each probe to HbA1c diminished after the removal of SEM observations.

### 3.3. Identification of HbA1c-Associated Polymorphic DNAm Loci

A total of 302,943 polymorphic probes were identified using the EPIC manifest file. Each probe was regressed against HbA1c values adjusted for age, sex and BMI. A total of 54 polymorphic probes were associated with HbA1c at an FDR < 5%. The top 30 ranked HbA1c-associated polymorphic probes are described in Table 3.

Of the 54 polymorphic DNAm probes, the island status of 11 were identified. The majority resided in shores (*n* = 7), with the remainder in subsequent islands (*n* = 3) and shelves (*n* = 1). The 54 probes mapped to 37 unique genes, with functional enrichment using UniProt for alternative splicing (FDR *p*-value = 0.0016). These genes also mapped to several relevant KEGG pathways, including type 1 diabetes mellitus, insulin secretion, PI3K-Akt signaling and diabetic cardiomyopathy.

### 3.4. Genome-Wide cg19693031 meQTL Analyses

Methylation quantitative trait loci (meQTL) analyses were conducted in both cis and trans to identify genetic factors that confound the methylation status of cg19693031. As underlying genomic mechanisms may differ according to proximity, our analyses were separated into: (1) cis meQTL (SNP-CpG distance < 1 Mb, *n* = 117), (2) long-range cis meQTL (>1 Mb intra-chromosomal, *n* = 38,750) and (3) trans meQTL (inter-chromosomal, *n* = 459,464). A total of 7 and 2052 meQTLs acting in cis and long-range cis were detected with nominal significance, with the degree of explained methylation variance ranging from 0.45% to 1.35% and 0.41% to 4.16%, respectively. While the cis long-range meQTLs explained more variance than those acting in cis, the magnitude of explained variance did not correlate with the SNP’s proximity to cg19693031. The meQTLs acting in cis are described in Table 4, while the top 30 long-range cis-meQTLs are described in Table 5. A total of 23,734 meQTLs acting in trans were also detected with nominal significance, with the degree of explained methylation variance ranging from 0.39% to 4.35%. Similar to those acting in cis, there were no observable differences between the chromosomes. The top 30 trans meQTLs are described in Table 6.

To determine the magnitude of meQTL effects on cg19693031 methylation, a multivariate linear regression model was built using meQTLs that demonstrated an R^2^ ≥ 0.03 (long-range cis (*n* = 5) and trans (*n* = 18)). Using an additive step-wise approach, genetic factors were ranked and fed into the model based on their adj R^2^. The associations between two meQTLs and cg19693031 methylation were no longer observed when incorporated into the model and were dropped from further analyses. The final meQTL model captured 43.23% of the variance of cg19693031 methylation (*p* < 2.2 × 10^−16^).

It has been previously suggested that some of the meQTL effects may in fact be occurrences of genetic and environmental interactions (GxE). To better understand the genetic and environmental confounding architecture influencing the methylation of cg19693031, we assessed whether additive or interactive effects explain the variability of cg19693031 methylation better than genetic factors alone. As a means of comparison, HbA1c was first regressed against cg19693031 methylation adjusted for age (R^2^ = 0.1314, *p* < 2.2 × 10^−16^). With the incorporation of HbA1c values to the final meQTL model (G + E), the amount of variance captured increased by 6% (R^2^ = 0.4968, *p* < 2.2 × 10^−16^). Using ANOVA, the additive G + E model significantly outperformed the genetic factors alone (*p* = 2.62 × 10^−14^).

Next, GxE analyses were conducted and compared to the G + E effects for each of the 21 retained meQTLs. Only one meQTL, rs74439, demonstrated a significant GxE term (*p* = 0.0146). The GxE effect explained ~ 1% more variance than the G + E model (R^2^ = 0.1712 vs. R^2^ = 0.1630) and demonstrated a significantly better fit (ANOVA: *p* = 0.0156). Incorporating the rs74439 GxE term to the final G + E meQTL model captured an additional 1% of the variance (Table 7: R^2^ = 0.5077, *p* < 2.2 × 10^−16^), and explained the variability of cg19693031 methylation better than genetic or additive G + E factors alone (ANOVA: *p* = 8.896 × 10^−16^ and *p* = 7.49 × 10^−4^, respectively).

### 3.5. Functional Mapping of meQTL SNPs

Next, we performed secondary analyses to better characterize the functional effects of genetic variation on cg19693031 methylation. The leading hypothesis to explain cis-meQTL effects is that, if a meQTL is located in a TFBS and hinders the TF from binding, the methylation of surrounding CpGs can be altered directly (recruitment of DNMT or TET enzymes) or indirectly.

Of the 25,792 meQTLs, a total of 21 had confirmed associations with T2D via genome-wide association studies. Additionally, a total of 986 are located within exons and 335 are missense mutations. The meQTL SNPs mapped to 4687 genes, with enrichment of two or more SNPs found within 2075 genes. These genes functionally map to several processes, including: alternative splicing (*p* = 2.09 × 10^−25^), ABC transporters (*p* = 1.7 × 10^−4^), insulin secretion (*p* = 8.1 × 10^−4^), lymphocyte mediated immunity (*p* = 2.61 × 10^−4^), immunoglobulin production (*p* = 1.19 × 10^−4^), complement activation (*p* = 2.50 × 10^−6^), immune system process (*p* = 2.32 × 10^−7^) and calcium ion transmembrane transporter activity (*p* = 6.23 × 10^−4^).

To gain insights into the molecular networks involved in nuclear regulation underlying the relationship between genetic variation and cg19693031 methylation, we assessed whether the proximal candidate gene at a trans-acting locus showed covariation with cg19693031 methylation. Using data from the eQTLGen Consortium (*n* = 31,684 samples), a total of 14,826 cis-eQTL associations with 5955 unique trans-meQTLs were identified with Bonferroni-corrected significance, with 3249 meQTL SNPs influencing the expression of 2+ genes. The identified cis-eQTLs mapped to 6861 unique genes, with enrichment of two or more cis-eQTLs influencing 3542 genes.

While the biological mechanisms underlying cis-meQTLs are easily understood and experimentally demonstrated, few clear examples for trans have been uncovered. The simplest hypothesis is that SNPs that act as eQTLs of global methylation regulators have downstream effects as trans-acting meQTLs. A similar potential mechanism is that an SNP residing in the coding region of methyl-specific-binding-proteins could alter their specificity and function and, therefore, modify the DNAm of their binding sites. To that end, several of the identified eQTL genes are known methylomic regulators, including: DNAm writers (DNMT1 and DNMT3a), readers (MBD2, MBD5, UHRF1, UHRF2 and BAZ2B) and erasers (TDF and SMUG1). Further, several of the eQTL genes are known regulators of TXNIP, including: transcription factors (FOXO1, HSF2, RXRA and KLF6), chromatin modifications (HDACs and NF-YA) and factors regulating mRNA turnover (mir-17, ZFP36, NEDD1, NEDD4, NEDD9, WWP1, SMURF2 and HECW2). Provided that distal residues can be brought into physical proximity by 3D structures, an SNP could affect DNAm levels at CpG sites in trans by acting either through cis-meQTL mechanisms or by disrupting the formation of structural loops. To that end, two of the three cohesion subunits were identified as an eQTL gene (SMC3 and RAD21).

**Figure 3:**
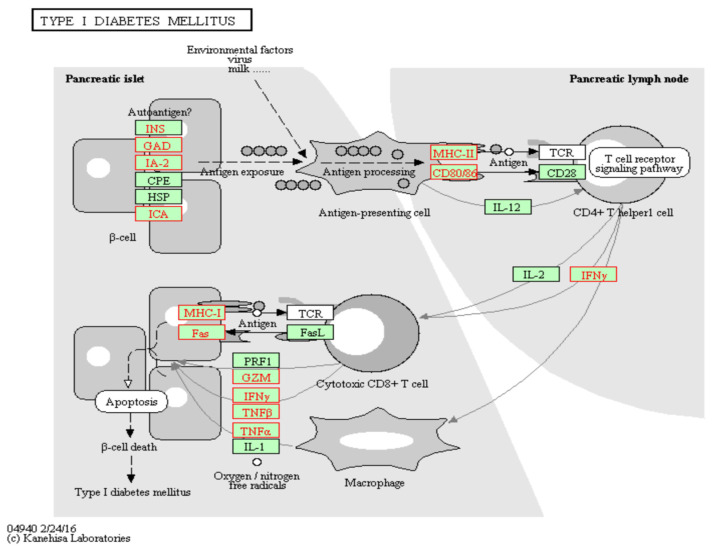
Cis e-QTL genes with trans-meQTL effects on cg19693031 identified in type 1 diabetes pathology. Cis e-QTL genes with meQTL effects on cg19693031 methylation are highlighted in red (*n* = 23).

**Figure 4:**
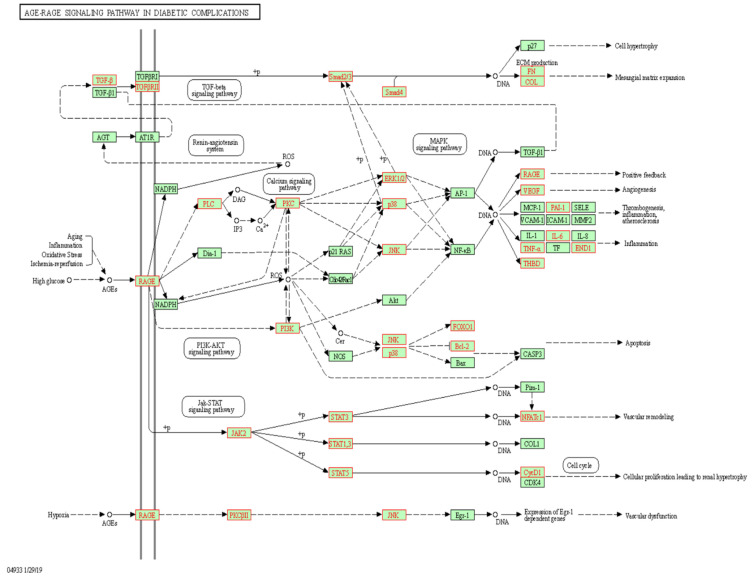
Cis e-QTL genes with trans-meQTL effects on cg19693031 identified in AGE-RAGE signaling in diabetic complications. Cis e-QTL genes with meQTL effects on cg19693031 methylation are highlighted in red (*n* = 34).

To gain insights into the potential role of trans meQTL effects on cg19693031 in the pleiotropic functions of TXNIP, functional gene mapping was conducted utilizing KEGG and GO. The eQTL genes mapped to key pathways that are associated with TXNIP’s pathophysiological role in diabetes and coronary heart disease, including: metabolic pathways (*n* = 504), PI3K-Akt signaling (*n* = 95), MAPK signaling (*n* = 81), cytokine-cytokine receptor interaction (*n* = 72), Rap1 signaling (*n* = 66), lipid and atherosclerosis (*n* = 65), ras signaling (*n* = 59), calcium signaling (*n* = 56), mTOR (*n* = 55), cAMP signaling (*n* = 52), diabetic cardiomyopathy (*n* = 49), insulin signaling pathway (*n* = 48), apoptosis (*n* = 46), Wnt signaling (*n* = 45), insulin resistance (*n* = 45), AMPK signaling (*n* = 45), FoxO signaling (*n* = 44), natural killer cell mediated cytotoxicity (*n* = 44), non-alcoholic fatty liver disease (*n* = 43), TNF signaling (*n* = 39), leukocyte transendothelial migration (*n* = 37), NK-kappa B signaling (*n* = 35), AGE-RAGE signaling pathway in diabetic complications (*n* = 34), TGF-β signaling (*n* = 33), b cell receptor signaling (*n* = 33), glucagon signaling (*n* = 27), type 1 diabetes (*n* = 23), PPAR signaling (*n* = 21), hypertrophic cardiomyopathy (*n* = 21), regulation of lipolysis in adipocytes (*n* = 18), glycolysis and gluconeogenesis (*n* = 18), insulin secretion (*n* = 18), ABC transporters (*n* = 17) and type 2 diabetes (*n* = 12). The eQTLs identified in type I diabetes and AGE-RAGE signaling in diabetic complications are illustrated in Figure 3 and Figure 4, respectively. TXNIP exerts its effects, in part, through the negative regulation of thioredoxin, which both TXN and TXN2 were identified as eQTL genes. Further, the eQTL genes also mapped to pathways associated with TXNIP’s pathophysiological role in cancer and neurodegenerative diseases, including: pathways in cancer (*n* = 159), pathways in neurodegeneration (*n* = 135), Alzheimer’s disease (*n* = 105), Huntington disease (*n* = 81), Parkinson’s disease (*n* = 68), microRNAs in cancer (*n* = 66), proteoglycans in cancer (*n* = 65), chemical carcinogenesis—ROS (*n* = 60), transcriptional regulation in cancer (*n* = 59), hepatocellular carcinoma (*n* = 52), Hippo signaling pathway (*n* = 50), cellular senescence (*n* = 49), breast cancer (*n* = 34) and p53 pathway (*n* = 29).

### 3.6. Genome-Wide cg19693031 GxE Analysis

To gain a better understanding of the genetically contextual effect of HbA1c levels on cg19693031 methylation, genome-wide GxE analyses were conducted. A total of seven and 1355 GxE terms acting in cis and long-range cis were detected with nominal significance, with the degree of explained methylation variance ranging from 13.31% to 14.05% and 12.32% to 16.67%, respectively. A total of 16,760 GxE terms acting in trans were detected with nominal significance, with the degree of explained variance ranging from 10.98% to 17.80%. Of the 18,797 GxE terms, a total of 964 were identified in our meQTL analyses (long-range cis: *n* = 81; trans: *n* = 883).

To determine the magnitude of GxE effects on cg19693031 methylation, an additive step-wise linear regression was fit using GxE terms that demonstrated an adj R^2^ ≥ 0.1550 and did not contain missing values (*n* = 44). Notably, a majority of the retained GxE terms were previously identified as meQTLs (*n* = 32). The features were ranked and fed into the model based on their adj R^2^, with retainment dependent on each feature remaining significant and a significant increase in the captured variance. A total of six GxE terms were retained for the final GxE model. The final model explained 27.78% of the variance of cg19693031 methylation (Table 8: *p* < 2.2 × 10^−16^), and significantly improved the fit compared to HbA1c alone (ANOVA: *p* < 2.2 × 10^−16^).

Next, we performed secondary analyses to better characterize the functional effects of the genetic variation of the GxE loci on cg19693031 methylation. Of the 18,797 SNPs, a total of nine had confirmed associations with T2D via genome-wide association studies. Additionally, a total of 681 are located within exons and 261 are missense mutations. The 18,797 GxE SNPs mapped to 3808 genes, with enrichment of two or more SNPs found within 1533 genes. Interestingly, these genes functionally mapped to alternative splicing (*p* = 1.183 × 10^−36^, the same as the HbA1c-associated polymorphic DNAm probes and meQTLs. Similar to the meQTLs, the GxE network mapped to several key TXNIP mechanistic pathways, including the cell adhesion (*p* = 1.74 × 10^−11^), glutamatergic synapse (*p* = 1.89 × 10^−5^), calcium signaling pathway (*p* = 1.7 × 10^−4^), insulin secretion (*p* = 1.6 × 10^−3^) and PI3K-Akt signaling pathway (*p* = 1.6 × 10^−3^).

### 3.7. Integrated Model of Genetic and Environmental Effects for cg19693031

Next, we assessed whether an integrated genetic and environmental model accounting for additive and interactive effects explains cg19693031 methylation variability better than either alone. Using a similar step-wise approach, a total of 20 G + E and 6 GxE terms were used to build the integrated model. The final integrated model captured 55.15% of the methylation variance (Table 9: *p* < 2.2 × 10^−16^) and performed significantly better than the genetic factors alone (ANOVA: 3.79 × 10^−8^).

### 3.8. cg19693031 Methylation Classifier for Predicting T2D Status

To determine the ability of cg19693031 to predict T2D status, a series of logistic regression classifiers were built, adjusting for age, sex and BMI. The covariate and cg19693031 models performed equally well in distinguishing diabetic from normoglycemic subjects (Table 10A: AUC = 0.72 and AUC = 0.73, respectively), and prediabetic from normoglycemic subjects (Table 10B: AUC = 0.52 and AUC = 0.53, respectively). However, the model incorporating cg1963031 performed better than the covariate model alone in distinguishing diabetic from prediabetic subjects (Table 10C: AUC = 0.68 and AUC = 0.72, respectively).

To determine if correcting for confounding local TXNIP genetic variation improves the predictive ability of the cg19693031 models for T2D status, a series of logistic regression classifiers were built, adjusting for age, sex and BMI. Three SNPS located within TXNIP that were previously identified as being associated with diabetes and cardiovascular disease were assessed. Of these, two were located near cg19693031 in the 3′UTR (rs7211 and rs7212), and one SNP was located in the 5′UTR. The incorporation of any local SNP or the addition of all three did not significantly improve the cg19693031 models for T2D status (Table 10).

### 3.9. Genome-Wide cg19693031 GxMeth Analyses for HbA1c

**Figure 5:**
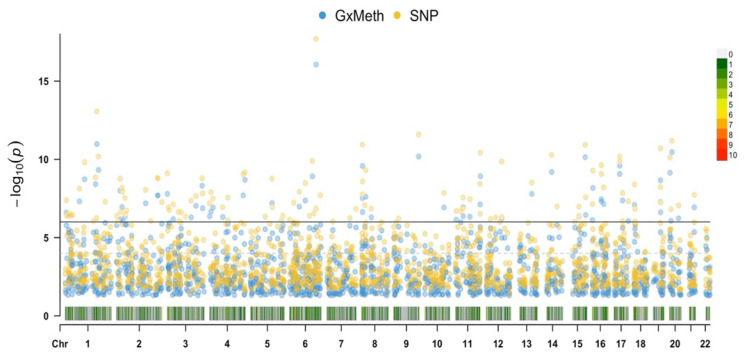
Manhattan plot illustrating the association of GxMeth and SNP terms with HbA1c. Results were adjusted for age, sex and BMI, and corrected for multiple comparisons. Associations at *p* < 1.33 × 10^−7^ are above the horizontal black line. The chromosome density, number of significant SNPs residing within a 1 Mb window, is located at the bottom of the plot and the color-coded legend is to the right.

Based on the understanding that trans genetic variation can confound DNA methylation status, we conducted a genome-wide analysis of SNP interactions with cg19693031 using the Multi-Ethnic Beadchip. The HbA1c values were regressed against additive and interactive GxMeth terms for each SNP, controlling for age, sex and BMI, with a total of 614,729 independent analyses conducted. After correcting for multiple comparisons, a total of 1476 and 1987 SNPs had significant GxMeth and additive effects, respectively (Figure 5). The top-25-ranked GxMeth SNPs are provided in Table 11.

**Figure 6:**
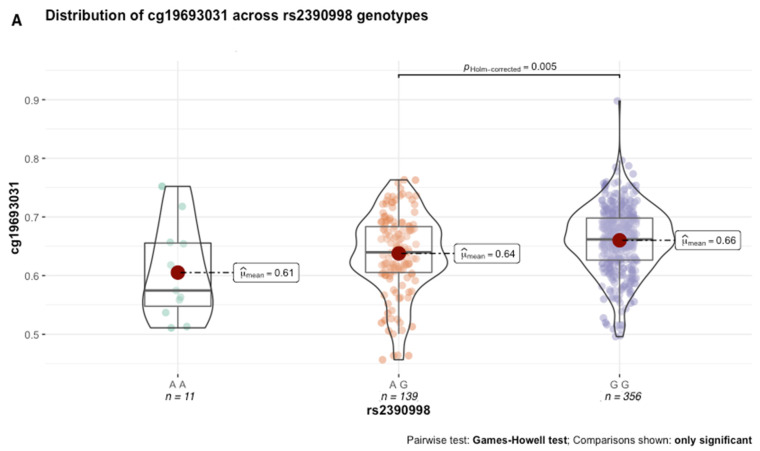

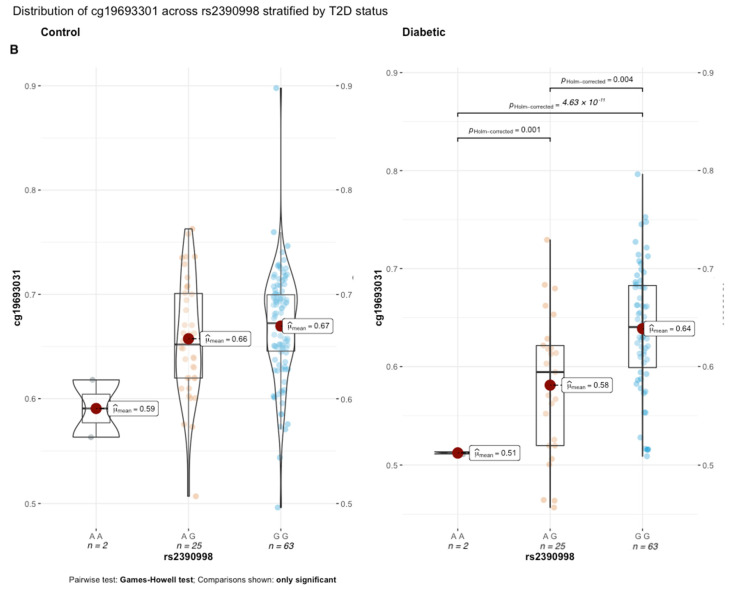
cg19693031 distribution as a function of rs2390998 genotype across all 506 subjects (**A**) and stratified by T2D status (**B**).

**Figure 7:**
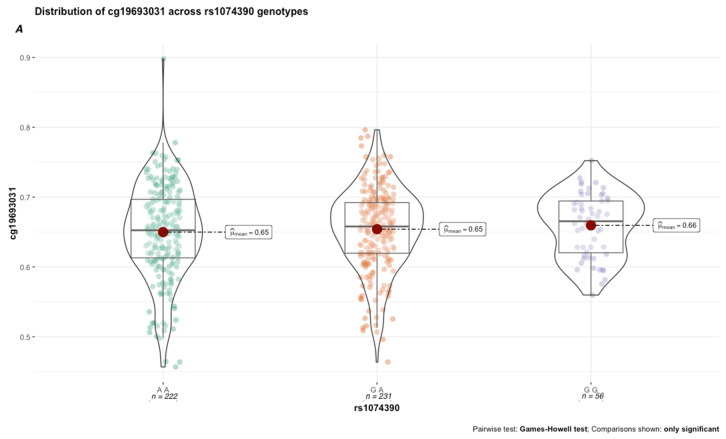

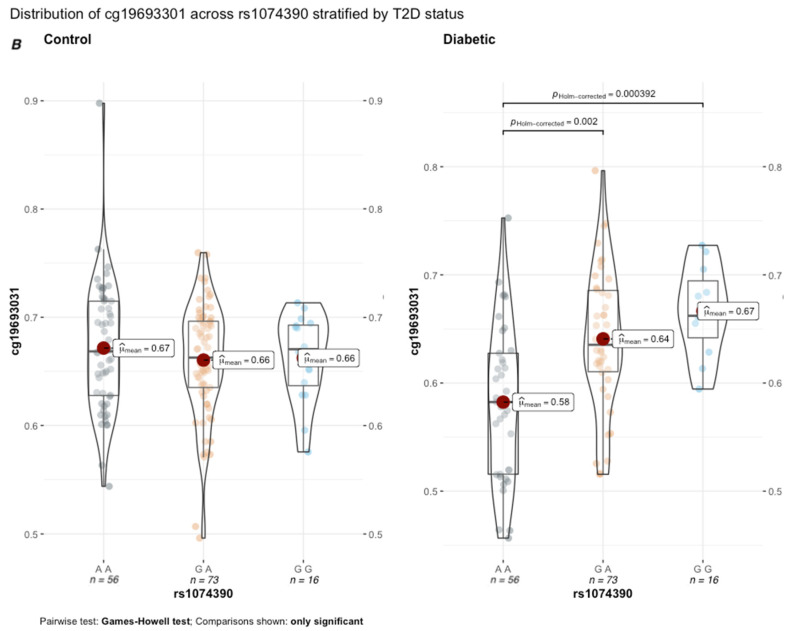
cg19693031 distribution as a function of rs1074390 genotype across all 506 subjects (**A**) and stratified by T2D status (**B**).

Next, we performed secondary analyses to better characterize the functional effects of genetic variation in the GxMeth loci on cg19693031 methylation. First, we conducted methylation quantitative trait loci (meQTL) analysis to determine the extent to which genetic variation in the GxMeth loci influences cg19693031 methylation. After Bonferroni correction, rs2390998 was identified as a meQTL (*p* = 0.045) with a small effect size (0.04, 95% CI = [0.01, 0.08]). As illustrated in Figure 6A, the average methylation across the 506 subjects was significantly lower in rs2390998 heterozygotes (AG) than major allele homozygotes (GG) (p_Holm-corrected_ = 0.005, Δ*β* = 2%). Notably, the genetic influence of rs2390998 on methylation was only prominent in diabetic subjects, with the most significant difference in methylation seen between the homozygote genotypes (Figure 6B; p_Holm-corrected_ = 4.63 × 10^−11^, Δ*β* = 13%; effect size = 0.16, 95% CI = [0.05, 0.32]). Next, we conducted a genetic x environment analysis (GxE) to determine the genetically contextual effect of HbA1c levels on cg19693031 methylation. After Bonferroni correction, rs1074390 had a significant GxE effect (*p* = 0.048; effect size = 0.165, 95% CI [0.11, 0.22]. While the average cg19693031 methylation did not significantly differ as a function of rs1074390 genotype across the cohort (Δ*β* = 1%), a significant difference was seen when stratified by T2D status (Figure 7). Similar to rs2390998, differences in methylation across the rs1074390 genotype were observed only in diabetic subjects, with the most significant difference in methylation observed between the homozygote genotypes (p_Holm-corrected_ = 3.92 × 10^−4^; Δ*β* = 9%; effect size = 0.38, 95% CI [0.12, 0.56].

**Figure 8:**
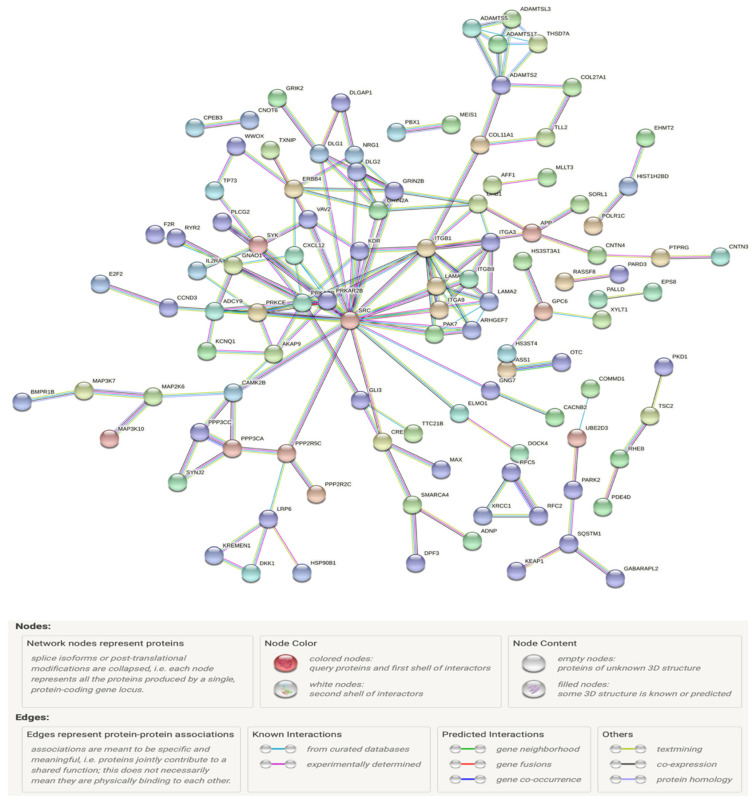
Protein–protein interactome (PPI) generated from regression analyses of significant cg19693031 GxMeth effects against HbA1c.

The significant GxMeth SNPs mapped to 560 unique genes, with enrichment of two or more SNPs found within 103 genes. Of those 560 genes, 167, 144 and 22 genes had confirmed associations to metabolic and cardiovascular disorders and type 2 diabetes, respectively [4,22]. To gain functional insights into these significant GxMeth interactions, a protein–protein interaction network (PPI) was generated using data from the STRING database. A total of 487 genes were correctly matched to the database. Figure 8 illustrates this network, with nodes only shown if they have an edge with a minimum interaction score of 0.9. This network consisted of 477 nodes with 158 edges (PPI enrichment *p* = 4.1 × 10^−9^), with an average node degree of 0.662. This network mapped to several biological process GO pathways that are highly associated with the development and complications of T2D, including regulation of voltage-gated sodium channel activity (*p* = 1.33 × 10^−2^), protein kinase C-activating G protein-coupled receptor signaling pathway (*p* = 1.26 × 10^−2^), cell communication involved in cardiac conduction (*p* = 1.41 × 10^−2^), cardiac muscle cell action potential involved in contraction (*p* = 3.65 × 10^−2^), regulation of heart rate (*p* = 6.63 × 10^−3^), calcium-mediated signaling (*p* = 2.30 × 10^−2^) and angiogenesis (*p* = 1.13 × 10^−2^). This network also mapped to several KEGG pathways, including glutamatergic synapse (*p* = 3.8 × 10^−3^), dilated cardiomyopathy (*p* = 7.5 × 10^−3^), Rap1 signaling pathway (*p* = 8.1 × 10^−3^), PI3K-Akt signaling pathway (*p* = 1.56 × 10^−2^), phospholipase D signaling pathway (*p* = 2.2 × 10^−2^), hypertrophic cardiomyopathy (*p* = 2.27 × 10^−2^) and Wnt signaling pathway (*p* = 2.42 × 10^−2^).

### 3.10. Integrated Genetic–Epigenetic Classifier for Predicting T2D Status

To determine if correcting for genetic factors with significant GxMeth effects improves the predictive ability of cg19693031 for T2D status, a series of balanced random forest (BRF) classifiers were built, adjusting for age, sex and BMI (Table 12). Using a repeated stratified k-fold cross-validation approach, each BRF classifier with random under-sampling was built to evaluate the features’ ability to discriminate between control and diabetic subjects. Similar to the logistic regression modeling, two base classifiers were built using the covariate variables solely and another incorporating cg19693031 (AUC = 0.68 and AUC = 0.71, respectively). Next, the 1476 GxMeth genetic factors were incorporated into the model, with a decrease in performance observed in comparison to the base models (AUC = 0.67). This decrease in performance is reflective of the random forest’s known vulnerability to overfitting due to correlated features with little discriminatory value.

**Figure 9:**
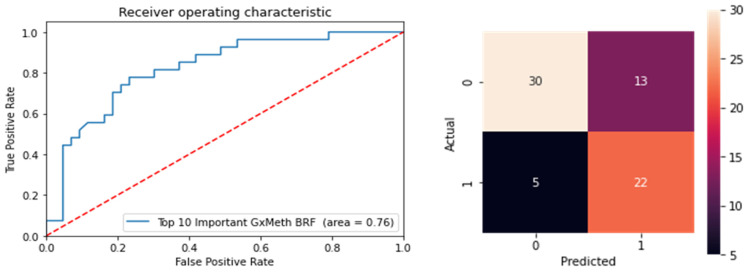
Testing of the GxMeth model using the top 13 ranked features for distinguishing normoglycemic controls and diabetic subjects.

To resolve this, we performed multivariate ensemble feature selection using random forest with a subsequent BRF classifier to reduce the dimensionality of the dataset. Multivariate approaches consider conditional higher-order interactions between several or all features simultaneously when measuring each feature’s relevance to the overall classification task. A total of 554 features were selected by the random forest learner, including the covariates, cg19693031 and 550 non-local GxMeth SNPs. Using a repeated stratified k-fold cross-validation approach with random under-samplings of the minority class to counteract class imbalance, a BRF model was built to evaluate the selected features for predicting control versus diabetic subjects. The performance of the model improved, with an AUC of 0.74. To further reduce the dimensionality of the dataset, we used the Gini importance to select the top-ranked relevant features. The selected features were ranked according to their Gini Importance, and the top 13 features were retained (Table 13). Using these features, a final BRF classifier was built. The classifier performed better than the previous models, with an AUC of 0.76 (81% sensitivity, 70% specificity). The confusion matrix and ROC curve are illustrated in Figure 9.

## 4. Discussion

Using data from a group of African American adults, we confirm and extend the prior findings, showing that the methylation of cg19693031 has a significant dose-dependent relationship with HbA1c. We identify confounding genetic variations influencing the methylation status of cg19693031 both independent of and in combination with HbA1c. The pathway analyses identified the regulatory mechanisms related to the 3D genomic structure and TXNIP’s role in disease susceptibility. Finally, we demonstrate that correcting for genetic confounding improves the ability of cg19693031 to detect type 2 diabetes. Collectively, our analyses suggest that the demethylation response of cg19693031 to sustained hyperglycemia in white blood cells may play a pivotal role in the regulatory network effects that underlie several clinical phenotypes, with insights into TXNIP’s pleiotropic functions for disease onset and progression.

To the best of our knowledge, our study is the first full integration of genetic and hyperglycemia effects on cg19693031 methylation, laying the groundwork for a more comprehensive understanding of the molecular mechanisms linking the methylation status of TXNIP’s 3′UTR to disease. Collectively, we identify 11,240 genes that participated in significant interactions. The enrichment and functional genomic analyses suggest the crosstalk between genetic variants and the demethylation response of cg19693031 may contribute to the dysregulation of gene expression seen in diabetes. For example, TXNIP has been shown to be induced by glucose and overexpressed in diabetic patients [23]. In addition, the TXNIP locus is complexly regulated by multilayered mechanisms, including transcriptional regulation, microRNA, mRNA stabilization and protein degradation [24]. Our analyses identified several of these regulators, including transcription factors (FOX01, HSF2, RXRA and KLF6) and modulators of mRNA stability (mir-17, ZFP36, NEDD1, NEDD4, NEDD9 and WWP1). In addition, several factors known to influence genome and epigenome plasticity were identified, including methylome regulators, histone modifiers and subunits of the cohesion complex. While our analyses suggest that the interplay between cg1963031 methylation and genetic variation may influence the transcriptional program of leukocytes in the context of hyperglycemia, external validation with expression data is needed.

Prior studies have suggested the utility of TXNIP as a biomarker for diabetes due to its hyperglycemia-induced overexpression and the strong association of cg19693031 with HbA1c [7]. While the demethylation of cg19693031 correlates to an increase in TXNIP transcription [5], this upregulation cannot be adequately explained by the demethylation of a 3′UTR CpG site as others have suggested. Whereas methylation within the CpG islands and shores near promoter regions correlates to transcriptional repression [25], the literature to support the same conclusion within gene bodies is lacking. Indeed, the recent work by Albao and colleagues suggests that the demethylation of cg19693031 promotes spurious transcription of the 3′UTR [26]. Prior studies have demonstrated the regulation of TXNIP via miRNA directed degradation of the mRNA, notably miR-17 [27]. Therefore, the spurious transcription of the 3′UTR intragenic region could potentially sequester the miRNA degradation by acting as a competitive endogenous RNA, resulting in an increase in TXNIP mRNA stability. Our findings further support the hypothesis that the demethylation response of cg19693031 may increase the mRNA stability of TXNIP. Further research is needed to validate and extend upon the role of the methylome in the regulation of TXNIP.

Our findings will be particularly useful for those seeking to better understand the pathophysiological function of TXNIP in order to optimize diabetes medicine. For example, several cg19693031 interacting genes were enriched in both type 1 (*n* = 27) and type 2 diabetes (*n* = 26). In addition, biological pathways related to diabetes were identified, including: insulin section, downstream signaling of insulin and glucagon and sensory perception of chemical stimulus. TXNIP emerged as a viable therapeutic target for diabetes due to its role in pancreatic β-cell apoptosis via activation of the mitochondrial death pathway [28]. The activation of the NLRP3 inflammasome results in the release of proinflammatory cytokines and apoptosis [29]. Critical to this report, TXNIP-induced inflammasome activation and IL-1β production are primarily observed in resident innate immune cells and not the islet β cells themselves [30,31,32]. Pathway analyses identified several mechanisms related to β-cell destruction, such as T-cell signaling and cytotoxic effects and ROS-induced apoptosis. Remarkably, strong interactions were observed with five major islet autoantigens (INS, GAD, IA-2, CPE and ICA). A total of 88 and 66 confounding genetic variants mapped to the NOD-like receptor signaling pathway and apoptosis. In particular, cg19693031 interacted with key loci for inflammasome activation (TRX, regulators of NLRP3 expression and NLRP3 itself), proinflammatory effects (IL-1β, IL-18, IL-6 and TNFα), death receptors (TRAIL-R, Fas and FADD) and other pro-apoptotic genes (BCL-2, BAD, JNK, BAK, ATM and PUMA). Further, TXNIP has been implicated in the development of diabetes-induced complications, including coronary heart disease. In this report, we identified loci mapping to lipids and atherosclerosis (*n* = 102), diabetic cardiomyopathy (*n* = 73), insulin resistance (*n* = 66) and AGE-RAGE diabetic complications (*n* = 59). This suggests that the pathophysiological role of the demethylation response of cg19693031 may generalize across the diabetes subtypes. As the diagnosis of diabetes mellitus is based on clinical presentation rather than specific molecular defects, overlaps in their etiologies have been documented. Indeed, prior studies have demonstrated the association of cg19693031 methylation and T1D. Further, current clinical trials are investigating the use of Verapamil for treatment of T1D, which has been shown to decrease the expression of TXNIP.

Altered TXNIP activity has been implicated in the development of several diseases aside from diabetes, including atherosclerosis, cancer and neurodegenerative diseases [33,34,35,36]. Our functional analyses identified enrichment in several of these diseases, including pathways in cancer (*n* = 274), pathways in neurodegeneration (*n* = 205), Alzheimer’s disease (*n* = 155), microRNAs in cancer (*n* = 106), transcriptional misregulation in cancer (*n* = 100) and the Hippo signaling pathway (*n* = 84). Moreover, prior studies have demonstrated an association between cg19693031 methylation and chylomicrons (Class A), triglycerides, hexose, α-hydrobutyrate, systolic hypertension, waist circumference, gender, metabolic syndrome, type 1 diabetes, coronary heart disease, breast cancer and colon cancer [7,37,38,39,40]. Taken together, this suggests that the demethylation response of cg19693031 in white blood cells is not specific to diabetes, let alone T2D. Therefore, the clinical use of cg19693031 methylation status in its current form as a biomarker for T2D diabetes may lead to inappropriate clinical decisions and poor patient outcomes. Extensive follow-up studies will be required to demonstrate the ability of cg19693031 methylation to accurately stratify diabetes risk in spite of the non-specific demethylation response.

We also note that, among those with HbA1c confirmed diabetes, only 57% had come to clinical attention, and less than 1% were on diabetic medications. This observation is reflective of the long asymptomatic phase of the disease, and health disparities seen in African American communities, resulting in a high rate of undiagnosed T2D. Additionally, control and pre-diabetic subjects reported a DM diagnosis, 3% and 9%, respectively, but they did not report the use of anti-diabetic pharmacological therapy. This discordance may be reflective of subjects reporting a pre-diabetic diagnosis, or lowered glucose levels due to lifestyle changes or bariatric surgery.

The findings of this study should be considered in the context of its limitations. First, it is important to note that the FACHS cohort is almost exclusively mature adult African American women. Further examinations using a larger number of subjects of other ethnicities, age groups and equal proportions of biological sexes will be required to demonstrate generalizability. Second, although the HbA1c test is the clinical gold standard, it is not sensitive to all pre-diabetes phenotypes [41]. Therefore, replicating our findings with other glycemic indices is needed. Third, the SNPs identified with having significant interactions with cg19693031 DNAm may not be the polymorphism driving the interaction; instead, it may be a tag SNP in full or partial equilibrium with another SNP that is driving the interaction. Additionally, there are known limitations to the Illumina EPIC arrays, such as cross-reactive and polymorphic probes, and β-value skewing due to the normalization process [42,43]. Hence, a more precise method for measuring cg19693031 DNAm, such as digital PCR, should be used to validate and extend our findings. Finally, cg19693931 methylation is confounded by multiple disease processes and cellular contexts. Therefore, while cg19693031 methylation may be sensitive to sustained hyperglycemia, it is not specific to diabetes.

In summary, we confirm and extend the prior findings of the demethylation response of cg19693031 to increasing HbA1c values in an African American cohort. This study is the first to extensively examine the genetic and hyperglycemia contextual effects on cg19693031 methylation, laying the groundwork for a better understanding of TXNIP’s pleiotropic pathophysiological effects in the development of disease. Future studies examining the extent of non-diabetes cellular-context-confounding cg19693031 methylation are needed to assess the feasibility for translation and potential clinical utility.

## Figures and Tables

**Table 1:** Clinical and demographic characteristics of subjects.

	Control	Pre-Diabetic	Diabetic
	*N* = 142	*N* = 274	*N* = 90
Age	45.3 ± 8.4	46.7 ± 7.3	52.1 ± 9.7
Gender			
Male	36 (25)	69 (25)	20 (22)
Female	106 (75)	205 (75)	70 (78)
Ethnicity			
African American	140 (99)	273 (99)	90 (100)
White	2 (1)	-	-
Hispanic	-	1 (1)	-
HbA1c (%)	5.34 ± 0.26	5.96 ± 0.21	8.02 ± 1.84
BMI	31.91 ± 8.45	33.73 ± 7.91	37.85 ± 9.53
Systolic BP ^†^	133.98 ± 21.92	134.15 ± 19.59	139.67 ± 23.65
Diastolic BP ^†^	83.46 ± 13.24	83.59 ± 13.25	84.06 ± 15.46
Self-Report Dx ^‡^			
Diabetes	-	22 (8)	51 (57)
Hypertension	12 (8)	43 (16)	17 (19)
Cardiac Event	1 (1)	2 (1)	1 (1)
Self-Report Rx *			
Diabetes	-	-	3 (3)
Blood Pressure	12 (8)	39 (14)	17 (19)
Cholesterol	-	2 (1)	1(1)

Mean ± standard deviation for continuous variables. N (%) for categorical variables. ^†^ BP represents blood-pressure (mm Hg). ^‡^ Dx represents diagnosis. * Represents treatment.

**Table 2:** Top 21 ranked HbA1c-associated DNAm loci.

Rank	CpG	Gene	Position	Island Status	SEM	*p*-Value
1	cg19693031	TXNIP	3′UTR	Open Sea	-	4.43 × 10^−12^
2	cg02458882	OPTN	5′UTR	Island	HMO	3.78 × 10^−7^
3	cg21804949			Open Sea	LMO	5.28 × 10^−7^
4	cg14955495	CASKIN	TSS1500	Island	HMO	2.16 × 10^−6^
5	cg18890830	GPX6	Body	Open Sea	LMO	2.86 × 10^−6^
6	cg22544867	NELFCD	TSS1500	N Shore	LMO	4.35 × 10^−6^
7	cg03577153	ZNF350	TS200	Open Sea	HMO	4.95 × 10^−6^
8	cg14277924	ATP10D	Body	Open Sea	LMO	8.16 × 10^−6^
9	cg02184744	ANKRD11	Body	Open Sea	LMO	3.35 × 10^−4^
10	cg14471895	FAM120AOS	Body	Island	HMO	5.30 × 10^−4^
11	cg06655623	RASGEF1A	TSS1500	Open Sea	HMO	8.09 × 10^−4^
12	cg14691530			S Shelf	LMO	9.90 × 10^−4^
13	cg13990141	FAMQ20A0S	Body	Island	HMO	8.62 × 10^−3^
14	cg22942097			Island	HMO	0.012
15	cg01105172			Open Sea	LMO	0.018
16	cg26823705	NBPF20	Body	Open Sea	HMO	0.020
17	cg24623376	IL2RA	Body	Open Sea	LMO	0.020
18	cg10536901	SLC25A24	5′UTR	Island	LMO	0.020
19	cg05922331			Open Sea	LMO	0.030
20	cg03916864	UNG	TSS1500	Island	HMO	0.037
21	cg12936627	PTTG1IP	TSS200	Island	HMO	0.037
22	cg25962218	LRGUK	Body	Open Sea	HMO	0.039
23	cg05055927			Open Sea	LMO	0.043

Top 23 significant DNAm loci associated with HbA1c after FDR correction for multiple comparisons.

**Table 3:** Top 30 polymorphic CpGs associated with HbA1c values.

Rank	CpG	Gene	SNP	*p*-Value
1	cg03422185	BCKDHA	rs145173140	0.0003
2	cg18909131	KIAA0427	rs77389586	0.0003
3	cg04608829	CYP4F12	rs73926867	0.0003
4	cg22228131	BCAR3	rs527794238	0.0004
5	cg27350255		rs541533080	0.0009
6	cg04291024	TRAPPC9	rs114893201	0.0009
7	cg00539624		rs534060211, rs116003679	0.0009
8	cg04272044	RAD50	rs73257757	0.0013
9	cg21808468	MCC	rs1822487	0.0014
10	cg08491197		rs76497600	0.0020
11	cg07873290		rs114434159	0.0020
12	cg26502489	SCN2B	rs78096838, rs569992626	0.0020
13	cg00888992	BRF1	rs116101636	0.0022
14	cg09208162	NDUFA10	rs138899326	0.0024
15	cg20494686		rs148229470, rs141139268	0.0025
16	cg22098375	GABBR1	rs556388914, rs564227738	0.0025
17	cg05614028		rs559329156	0.0033
18	cg19804132	CCL8	rs181302524	0.0045
19	cg15166058	ACTG2	rs150940664	0.0047
20	cg23511885		rs62219339	0.0051
21	cg14264512	AKAP9	rs540981223	0.0054
22	cg18049287	ZBP1	rs532936093	0.0062
23	cg16018596		rs78999282	0.0068
24	cg23879504	PRLR	rs538603339, rs79823771	0.0077
25	cg01704651	C1ORF167	rs189737870	0.0083
26	cg25921170	ABL2	rs553415841	0.0097
27	cg23576118	RGS6	rs36322	0.0137
28	cg21442599	SMIM8	rs181909564, rs187248940	0.0137
29	cg19532212	TEX14	rs185842772	0.0142
30	cg21606577	RSPRY1	rs141350118	0.0142

Top 30 ranked significant polymorphic CpG loci associated with HbA1c after FDR correction for multiple comparisons.

**Table 4:** Cis-meQTLs nominally associated with cg19693031.

Rank	SNP	Gene	Distance	Adj R^2^	*p*-Value
1	rs587743199	NOTCH2NLA	2.26 × 10^5^	0.01353	0.003
2	rs16827006	ITGA10	9.83 × 10^4^	0.01028	0.008
3	rs16827043		4.61 × 10^4^	0.009199	0.011
4	rs61741868	ITGA10	9.99 × 10^4^	0.005841	0.030
5	rs10910843	RNF115	2.50 × 10^5^	0.005217	0.036
6	rs112006139	PDE4DIP	4.87 × 10^5^	0.004855	0.037
7	rs5617144	ANKRD35	1.14 × 10^4^	0.004491	0.045

**Table 5:** Top 30 long-range cis-meQTLs nominally associated with cg19693031.

Rank	SNP	Gene	Distance	Adj R^2^	*p*-Value
1	rs17023177	PTPN14	6.93 × 10^7^	0.0416	1.52 × 10^−6^
2	rs12718444	SLC2A1	1.02 × 10^8^	0.0324	1.86 × 10^−5^
3	rs2086856	SLC2A1	1.02 × 10^8^	0.0309	2.80 × 10^−5^
4	rs6702764		9.92 × 10^7^	0.0308	2.87 × 10^−5^
5	JHU_1.230641162		8.52 × 10^7^	0.03	3.57 × 10^−5^
6	rs34247575	PTGER3	7.41 × 10^7^	0.0268	8.58 × 10^−5^
7	JHU_1.19366997		1.26 × 10^8^	0.0267	8.80 × 10^−5^
8	rs74121148		1.19 × 10^7^	0.0266	9.06 × 10^−5^
9	rs2792599		1.02 × 10^8^	0.0253	1.30 × 10^−4^
10	rs9326132		1.02 × 10^8^	0.0253	1.31 × 10^−4^
11	JHU_1.78608038		6.68 × 10^7^	0.025	1.43 × 10^−4^
12	rs57013566		9.97 × 10^7^	0.0253	1.61 × 10^−4^
13	rs12091692		5.29 × 10^7^	0.0234	2.22 × 10^−4^
14	rs72895742	TTC22	9.02 × 10^7^	0.0229	2.52 × 10^−4^
15	rs6699702	PRDX1	9.95 × 10^7^	0.0229	2.52 × 10^−4^
16	rs2422139	C1orf105	2.69 × 10^7^	0.0227	2.63 × 10^−4^
17	rs10911734		3.40 × 10^7^	0.0227	2.67 × 10^−4^
18	JHU_1.45997600		9.94 × 10^7^	0.0225	2.85 × 10^−4^
19	JHU_1.992041		1.40 × 10^8^	0.0227	3.47 × 10^−4^
20	JHU_1.46359270	MAST2	9.91 × 10^7^	0.0209	4.35 × 10^−4^
21	rs2280511	GLIS1	9.15 × 10^7^	0.0208	4.50 × 10^−4^
22	JHU_1.90440644		5.50 × 10^7^	0.0203	5.04 × 10^−4^
23	JHU_1.49307071	AGBL4	9.61 × 10^7^	0.0203	5.04 × 10^−4^
24	JHU_1.26626016	UBXN11	1.19 × 10^8^	0.0203	5.16 × 10^−4^
25	rs78580993		3.99 × 10^7^	0.0202	5.18 × 10^−4^
26	exm111496	FCRL5	1.21 × 10^7^	0.0202	5.29 × 10^−4^
27	rs10864087		6.89 × 10^7^	0.0201	5.39 × 10^−4^
28	rs12033260	LOC101927244	7.42 × 10^7^	0.0201	5.43 × 10^−4^
29	rs3935008	PTPRVP	5.67 × 10^7^	0.02	5.47 × 10^−4^
30	rs11265240		1.41 × 10^7^	0.0199	5.69 × 10^−4^

**Table 6:** Top 30 distal meQTLs nominally associated with cg19693031.

Rank	SNP	Gene	Chromosome	Adj R^2^	*p*-Value
1	rs181228225		15	0.0425	1.30 × 10^−6^
2	rs74470648		3	0.0418	1.44 × 10^−6^
3	rs296200		5	0.0376	4.46 × 10^−6^
4	rs2390998		13	0.0366	5.86 × 10^−6^
5	rs744439		14	0.0364	6.33 × 10^−6^
6	JHU_5.106093036		5	0.0358	8.38 × 10^−6^
7	rs1054013	MEG3	14	0.0346	1.02 × 10^−5^
8	exm-rs11177669		12	0.0344	1.09 × 10^−5^
9	rs7330858		13	0.0338	1.30 × 10^−5^
10	rs1451452		2	0.0337	1.30 × 10^−5^
11	JHU_18.63134356		18	0.0336	1.38 × 10^−5^
12	JHU_16.9476218		16	0.0328	1.69 × 10^−5^
13	rs117580705		11	0.0326	1.74 × 10^−5^
14	exm-rs11221332	ETS1	11	0.032	2.09 × 10^−5^
15	rs1380989	FGF12	3	0.0315	2.43 × 10^−5^
16	rs331581		5	0.0304	3.24 × 10^−5^
17	rs3794207	CAMKK2	12	0.0302	3.38 × 10^−5^
18	rs568941		16	0.0297	3.93 × 10^−5^
19	rs7142470	LINC00520	14	0.0296	3.97 × 10^−5^
20	exm-rs2523608	HLA-B	6	0.0296	4.01 × 10^−5^
21	rs266273		2	0.0294	4.24 × 10^−5^
22	JHU_15.87493750	AGBL1	15	0.0295	4.28 × 10^−5^
23	rs73392704		14	0.0292	4.50 × 10^−5^
24	exm523653	POM121L2	6	0.0291	4.57 × 10^−5^
25	rs6887332	CDX1	5	0.029	4.75 × 10^−5^
26	JHU_14.23027963		14	0.0289	4.85 × 10^−5^
27	JHU_3.16412493		3	0.0312	4.98 × 10^−5^
28	rs11793972	FAM155A	9	0.0288	5.94 × 10^−5^
29	rs4442648		13	0.0285	5.43 × 10^−5^
30	rs73986979	FIBCD1	17	0.0282	5.89 × 10^−5^

**Table 7:** Association estimates for features in final multivariate G + E meQTL regression model with one GxE term for cg19693031.

Feature	β	*p*-Value
A1C	−0.021	***
rs181228225	0.024	***
rs11221332	−0.021	***
JHU_18.63134356	0.017	***
rs17023177	0.012	***
rs2390998	0.013	***
rs744439:A1C	0.008	***
rs6702764	−0.014	***
rs74470648	0.011	**
rs3794207	−0.012	**
rs331581	−0.012	**
JHU_1.230641162	−0.009	**
rs1451452	−0.011	**
rs7330858	0.018	**
rs117580705	0.018	**
JHU_5.106093036	−0.010	**
rs744439	−0.044	**
rs1380989	0.009	**
rs296200	0.009	**
rs12718444	−0.012	**
JHU_16.9476218	0.012	*
rs11177669	−0.007	*
rs1054013	−0.013	*

*p*-value significant codes: <0 (***), <0.001 (**), <0.01 (*). Overall model performance: adj R^2^ = 0.5077 and *p*-value < 2.2 × 10^−16^.

**Table 8:** Association estimates for features in final multivariate GxE regression model for cg19693031 methylation.

Feature	β	*p*-Value
rs2235338	0.059	**
rs72914579	−0.091	**
A1C:rs2235338	−0.008	**
JHU_1.52062655	0.082	**
rs11265240	0.058	**
A1C:rs72914579	0.011	*
JHU_2.234824901	0.054	*
A1C:JHU_1.52062655	−0.011	*
rs744439:A1C	0.007	*
A1C:JHU_2.234824901	−0.008	*
A1C:rs11265240	−0.007	*
rs744439	−0.027	
A1C	0.007	

*p*-value significiant codes: < 0.001 (**), < 0.01 (*). Overall model performance: adj R^2^ = 0.2778 and *p*-value < 2.2 × 10^−16^.

**Table 9:** Association estimates for features in final multivariate integrated additive and interactive genetic and HbA1c regression model for cg19693031 methylation.

Feature	β	*p*-Value
rs181228225	0.023	***
rs11221332	−0.019	***
JHU_18.63134356	0.017	***
rs744439:A1C	0.009	***
rs74470648	0.010	**
rs744439	−0.050	**
rs11177669	−0.009	**
rs1451452	−0.011	**
JHU_1.230641162	−0.008	**
rs11265240	0.052	**
rs17023177	0.011	**
rs117580705	0.018	**
rs6702764	−0.012	**
JHU_1.52062655	0.072	**
rs1054013	−0.014	**
rs7021911	0.138	**
A1C:rs7021911	−0.023	**
rs12718444	−0.012	**
rs3794207	−0.010	**
rs296200	0.009	**
rs7330858	0.015	*
rs331581	−0.009	*
rs1380989	0.008	*
A1C:rs11265240	−0.007	*
A1C:JHU_1.52062655	−0.010	*
rs62437200	−0.063	*
rs2390998	0.009	*
JHU_5.106093036	−0.008	*
rs7789476	−0.007	*
A1C:rs62437200	0.009	*
JHU_16.9476218	0.009	*
A1C	0.036	

*p*-value significant codes: <0 (***), <0.001 (**), <0.01 (*). Overall model performance: adj R^2^ = 0.5515 and *p*-value < 2.2 × 10^−22^.

**Table 10:** Performance metrics of logistic regression models for predicting normoglycemic vs. diabetic subjects (**A**), normoglycemic vs. prediabetic subjects (**B**) and prediabetic vs. diabetic subjects (**C**).

**Model ^†^**	**Predictors**	**Precision**	**Sensitivity**	**Specificity**	**AUC**
1	Age + Sex + BMI	0.63	70%	74%	0.72
2	Model 1 + cg19693031	0.66	70%	76%	0.73
3	Model 2 + rs7211	0.65	74%	74%	0.74
4	Model 2 + rs7212	0.66	70%	76%	0.73
5	Model 2 + rs9245	0.61	63%	74%	0.68
6	Model 2 + rs7211 + rs7212 + rs9245	0.66	70%	76%	0.73
^†^ Missing values for predictors result in analysis sample size of 227 subjects for all models.					
(**A**)					
**Model ^†^**	**Predictors**	**Precision**	**Sensitivity**	**Specificity**	**AUC**
1	Age + Sex + BMI	0.68	58%	46%	0.52
2	Model 1 + cg19693031	0.68	59%	46%	0.53
3	Model 2 + rs7211	0.7	57%	54%	0.55
4	Model 2 + rs7212	0.68	59%	46%	0.53
5	Model 2 + rs9245	0.68	61%	44%	0.52
6	Model 2 + rs7211 + rs7212 + rs9245	0.68	61%	44%	0.52
^†^ Missing values for predictors result in analysis sample size of 397 subjects for all models.					
(**B**)					
**Model ^†^**	**Predictors**	**Precision**	**Sensitivity**	**Specificity**	**AUC**
1	Age + Sex + BMI	0.43	67%	69%	0.68
2	Model 1 + cg19693031	0.51	67%	78%	0.72
3	Model 2 + rs7211	0.51	67%	78%	0.72
4	Model 2 + rs7212	0.5	63%	78%	0.71
5	Model 2 + rs9245	0.53	67%	79%	0.73
6	Model 2 + rs7211 + rs7212 + rs9245	0.46	59%	76%	0.67
^†^ Missing values for predictors result in analysis sample size of 350 subjects for all models.					
(**C**)					

**Table 11:** Top 25 ranked SNPs with GxMeth effects after Bonferroni correction.

Rank	SNP	Chr	Gene	MAF	GxMeth *p*-Value	SNP *p*-Value
1	rs1932189	6		0.027	8.85 × 10^−17^	1.98 × 10^−18^
2	rs10797745	1	DNM3	0.034	1.04 × 10^−11^	8.91 × 10^−14^
3	rs6110333	20	MACROD2	0.062	3.44 × 10^−11^	8.52 × 10^−12^
4	rs11244376	9		0.025	6.64 × 10^−11^	2.57 × 10^−12^
5	rs4369634	15		0.316	7.32 × 10^−11^	1.19 × 10^−11^
6	rs28904	17	SPACA3	0.084	2.56 × 10^−10^	6.76 × 10^−11^
7	rs2816490	8		0.158	2.65 × 10^−10^	1.17 × 10^−11^
8	rs78845567	1	XPR1	0.015	4.78 × 10^−10^	6.70 × 10^−11^
9	rs77998701	14		0.036	6.45 × 10^−10^	5.23 × 10^−11^
10	rs1764995	20		0.477	7.13 × 10^−10^	7.68 × 10^−11^
11	rs62068262	17		0.059	8.54 × 10^−10^	1.43 × 10^−10^
12	rs117219196	11		0.027	1.20 × 10^−9^	3.79 × 10^−11^
13	rs12204978	6		0.059	1.24 × 10^−9^	1.28 × 10^−10^
14	rs74635854	1		0.012	1.86 × 10^−9^	1.53 × 10^−10^
15	rs72711488	4		0.15	2.02 × 10^−9^	6.88 × 10^−10^
16	rs138418725	19	MAP3K10	0.014	2.18 × 10^−9^	1.98 × 10^−11^
17	rs16840887	1		0.023	3.85 × 10^−9^	1.46 × 10^−9^
18	rs62290693	3		0.269	4.84 × 10^−9^	1.65 × 10^−9^
19	rs1296720	16	CREBBP	0.032	6.70 × 10^−9^	5.26 × 10^−10^
20	rs17703186	2		0.095	9.96 × 10^−9^	1.72 × 10^−9^
21	rs66462704	3		0.079	1.60 × 10^−8^	7.75 × 10^−10^
22	rs142518718	13		0.013	1.61 × 10^−8^	3.10 × 10^−9^
23	rs116519465	4		0.013	2.00 × 10^−8^	9.16 × 10^−10^
24	rs16859517	2	NHEJ1	0.013	2.02 × 10^−8^	1.59 × 10^−9^
25	rs12676638	8		0.015	2.45 × 10^−8^	4.84 × 10^−10^

Abbreviations: Chr, chromosome; MAF, minor allele frequency. Top 25 SNPs based on the Bonferroni corrected *p*-value of the Gxmeth term in regression analyses against HbA1c values adjusted for age, sex and BMI.

**Table 12:** Balanced random forest models performance metrics for predicting normoglycemic vs. diabetic subjects.

Model	Predictors	Precision	Sensitivity	Specificity	AUC
1	Age + Sex + BMI	0.56	74%	63%	0.68
2	Model 1 + cg19693031	0.59	74%	67%	0.71
3	Model 2 + 1476 GxMeth SNPs	0.54	70%	63%	0.67
4	Model 2 + 551 selected GxMeth SNPs	0.69	67%	81%	0.74
5	Model 2 + Top 10 GxMeth SNPs	0.63	81%	70%	0.76

**Table 13:** Top ranked important features selected by a BRF for predicting normoglycemic vs. diabetic subjects.

Rank	Feature
1	Age
2	cg19693031
3	BMI
4	rs78125109
5	rs9311874
6	rs79244502
7	rs10496366
8	rs10496731
9	rs10032200
10	rs77173725
11	rs2227818
12	rs440617
13	rs3218194

## Data Availability

All data used in this study are available from the corresponding author on reasonable request.
